# Si-Miao-Yong-An (SMYA) Decoction May Protect the Renal Function Through Regulating the Autophagy-Mediated Degradation of Ubiquitinated Protein in an Atherosclerosis Model

**DOI:** 10.3389/fphar.2020.00837

**Published:** 2020-07-02

**Authors:** Ze-Bing Zhu, Ke Song, Wei-Jun Huang, Hui Li, Hui Yang, Yun-Qi Bai, Ke-Ting Guo, Rui-Bing Yang, Wen-Jiao Lou, Chen-Hui Xia, Bo Nie, Wei-Jing Liu

**Affiliations:** ^1^ Key Laboratory of Chinese Internal Medicine of Ministry of Education and Beijing, Dongzhimen Hospital Affiliated to Beijing University of Chinese Medicine, Beijing University of Chinese Medicine, Beijing, China; ^2^ Renal Research Institute of Beijing University of Chinese Medicine, Dongzhimen Hospital Affiliated to Beijing University of Chinese Medicine, Beijing, China

**Keywords:** autophagy, atherosclerosis, Si-miao-yong-an, traditional Chinese medicine, ubiquitinated protein

## Abstract

Hyperlipidemia is common, and its renal toxicity has attracted a great deal of attention. Si-miao-yong-an (SMYA) is a famous ancient decoction of traditional Chinese medicine (TCM), which is still widely used in clinical treatment. In this study, we observed and explored its efficacy and mechanism in protecting renal function in an atherosclerosis model. The results showed that the serum, Cr urinal KIM-1, and NGAL were significantly decreased in SMYA group. Although SMYA failed to alleviate the lipid accumulation, decrease p-NFκB, or increase SOD in kidney tissue, the levels of ubiquitinated protein and P62 were decreased in SMYA group. What is more, a higher LC3 II level was observed in the SMYA group. In conclusion, these data indicated that SMYA decoction may protect renal function in hyperlipidemia *via* regulating the autophagy-mediated degradation of ubiquitinated protein.

## Introduction

Hyperlipidemia is caused by a lipid over intake and/or metabolism disorder, which can result in not only atherosclerosis but also chronic kidney disease (CKD) (Ruan et al., 2009). Because of the prevalence of hyperlipidemia, the renal injury caused by hyperlipidemia has drawn lots of the world’s attention. Studies have indicated that mesangial cells, podocytes, and proximal tubule cells are involved in the hyperlipidemia-related renal injury. Among the three renal cells, proximal tubule cells seem to be more susceptible to lipid toxicity (Mandel, 1985; Adeosun et al., 2018). Hyperlipidemia can lead the accumulation of lipid in kidney cells, which results in over-production of reactive oxygen species (ROS) and activation of inflammation pathways. Excess ROS and inflammatory factor cause a negative effect to renal cells in the end (Gai et al., 2019).

Even though some mechanisms of hyperlipidemia-related CKD have been uncovered, there is little medicine that has proven to be effective in protecting renal function except for the allopathic therapeutic agents. However, Chinese herbs have been widely used in treating hyperlipidemia-related CKD in clinical practice. Several Chinese herbs have been proved to be beneficial to hyperlipidemia-related renal injury (Jiang et al., 2018; Liao et al., 2019). SMYA decoction is a famous ancient decoction of TCM. It was first recorded the book named “Hua tuo shen yi mi zhuan,” which was written during the Han dynasty. Even though this decoction has a long history, it is still widely used in China. In this study, we observed its efficacy in protecting renal function in an atherosclerosis model and explore its mechanism based on the autophagy-mediated degradation of ubiquitinated protein.

## Materials and Methods

### Animals

Male (aged between 6 and 8 weeks) ApoE-/- C57BL/6J mice and ApoE^f/f^ mice from the same litters were purchased from Beijing HFK bioscience CO., LTD, weighing 18–22g. All the mice were kept in a SPF animal house with a food and water freely available, 12 h light/dark, and a constant temperature environment of 24°C. The study followed the national guidelines for laboratory animal welfare and was approved by the Animal Ethics Committee of Beijing University of Chinese Medicine (NO.BUCM-4-2015071701-3001). All the mice were sacrificed under anesthesia.

### Preparation of SMYA Decoction

The SMYA Decoction consists of honeysuckle, radix scrophulariae, angelica sinensis, and liquorice. Four of the herbs above were boiled two times with pure water (the first time for 2 hours and the second time for 1 hour). The two decoctions mixed and concentrated to 2g/ml (The ratio of honeysuckle, radix scrophulariae, angelica sinensis, and liquorice was 3:3:2:1). The drug dose for mice was 9.1 times that for human (honeysuckle 90g, radix scrophulariae 90g, angelica sinensis 60g, and liquorice 30g) and calculated according to the body weight (the *Methodology of pharmacological experiment* edited by Professor Shuyun Xu). For example, a human needs 90g honeysuckle per 80Kg body weight. Then, a mouse needs 0.10g/10g body weight honeysuckle, which means 0.30g/10g body weight SMYA (0.10g honeysuckle, 0.10g radix scrophulariae, 0.07g angelica sinensis, and 0.03g liquorice).

### Establishment of Atherosclerosis Model

The atherosclerosis model was established by a high-fat diet and carotid cannulation surgery in the ApoE^-/-^ mouse. The surgery was operated after a 3-day adaptive feeding and 2-week high-fat feeding (containing15% fat and 0.25% cholesterol). Firstly, all apoE^-/-^ mice were fasted for 12 hours. After anesthesia, the right common carotid artery was exposed, and a silicone cannula (length: 2.5mm, inner diameter: 0.3mm) was fixed around the carotid artery (external diameter: about 0.5mm). Penicillin was injected intraperitoneally for 3 consecutive days after surgery to prevent infection.

### Treatment Administration

The atherosclerosis mice were randomly divided into two group—the model group and the SMYA group—and wild-type C57BL/6J mice (ApoEf/f) were used as the control—a blank group. The mice in SMYA group received a high-fat diet plus SMYA decoction, and the mice in model group received high-fat diet plus purified water, while the mice in blank group received ordinary diet plus purified water. The medicine or purified water was given by garage (0.15ml/10g) for 8 weeks.

### Biochemical Indicators Assay

After an 8-week intervention of SMYA decoction, blood was taken by removing eyeball after fasting for 8 h, and 6-hour urine was also collected. GLU, TC, TG, HDL, LDL, Scr, UREA, ALT, and AST of serum (some results were submitted as **Supplementary Materials**) were detected by automatic biochemical analyzer (AU5800, Beckman Coulter Co., Ltd.), and urinal NGAL and KIM-1 were detected with ELISA kits (NGAL ELISA kit, ab199083, abcam; KIM-1 ELISA kit, ab213477, abcam).

### Western Blot Analysis

Western blot analysis was performed as described previously (Liu et al., 2012). The primary antibodies against LC3B (dilution 1:1000, ab51520, abcam), SQSTM1 protein (dilution 1:1000, ab56416, abcam), p-NFκB (dilution 1:500, sc136548, santa cruz), MnSOD (dilution 1:5000, ab13533, abcam), and HRP-conjugated secondary goat antibodies (dilution 1:5000, SA00001-1 and SA00001-2; Proteintech) were used.

### Histopathology Study

For a histopathology study, the kidney tissue was fixed in 4% paraformaldehyde for 24h and was embedded with paraffin after gradient-alcohol dehydration, xylene vitrification, and waxdip. Sections that were 3 μm thick were used in HE staining, Masson staining, oil red staining, and the immunochemistry study. For the immunofluorescence study, the paraformaldehyde-fixed kidney tissue was embedded with an optimal cutting temperature compound and quickly frozen in the -20°C refrigerator. Sections that were 5 μm thick were used in the immunofluorescence study. The immunochemistry kit (PV-9005, ZSGB-Bio) was used in the immunochemistry study. The process is described briefly as follows: (a) Dewaxing with xylene, gradient-alcohol hydration, and antigen retrieval with citrate solution in microwave stove; (b) inactivation of peroxidase with the 3% hydrogen peroxide and blocking with goat serum; (c) incubated over night at 4°C refrigerator with first antibody (P62 antibody: dilution 1:500, ab56416, abcam; UB antibody: dilution 1:500, ab134953, abcam); (d) incubated for 30 min at 37°C with second antibody; (e) DAB coloration, hematoxylin staining, conventional dehydration, xylene vitrification. and sealing with gelatin; and (f) images were captured with microscope and analyzed with Image-pro plus 6.0 or scored by two researchers separately.

The process of immunofluorescence is described briefly as follows: (a) Antigen retrieval with citrate solution in microwave stove; (b) membrane penetration with 0.2% PBST for 20 min; (c) blocking with 5% donkey serum for 30 min in 37°C (d) incubated over night at 4°C refrigerator with first antibody (LC3B: dilution 1:1000, ab51520, abcam); (e) incubated for 1h at room temperature with second antibody (Alexa Fluor^®^ 488 donkey anti-rabbit IgG (H+L): dilution 1:2000, lot 1927937, Invitrogen by thermos Fisher Scientific); (f) nuclear was stained with DAPI (ZLI-9557, ZSGB-Bio); and (g) images were captured with fluorescence microscope and scored by two researchers separately.

### Oil Red O Staining

Oil red O dye (G1260, Solarbio life sciences) was diluted with distilled water (3:2) and was filtered before use. The process was briefly described as follows: (a) tissue soaked in the 60% isopropanol for 30s; (b) tissue stained with diluted Oil red O dye for 15min. (c) tissue soaked in the 60% isopropanol for 10s and then washed with distilled water; and (d) hematoxylin staining and tissue being sealed with glycerin gelatin.

### Statistical Analysis

Graphpad prism 6.01 (GraphPad Software, USA) was used in the Statistical analysis. Data were expressed as mean ± standard error ($\bar{x}$ ± SE). Normally distributed data were analyzed with one-way ANOVA, while non-normal distributed data were analyzed with nonparametric tests. If the P-value was less than 0.05, the statistical differences were considered to be significant.

## Results

### Establishment of Atherosclerosis Model

The HE staining, MASSON staining, and carotid ultrasound were used to evaluate the establishment of atherosclerosis model. In the model group, carotid plaque was observed. Foam cells could be seen under the intima in the HE staining. Results of MASSON staining showed that more collagen fiber expressed. Besides, the model mice had narrower carotid cavity and faster blood flow velocity, which detected by the ultrasound system (Vevo2100, FUJIFLM, America). All the results has been published in our previous paper (Ke, 2019).

### SMYA Decoction Could Protect Renal Function

**Figure 1:**
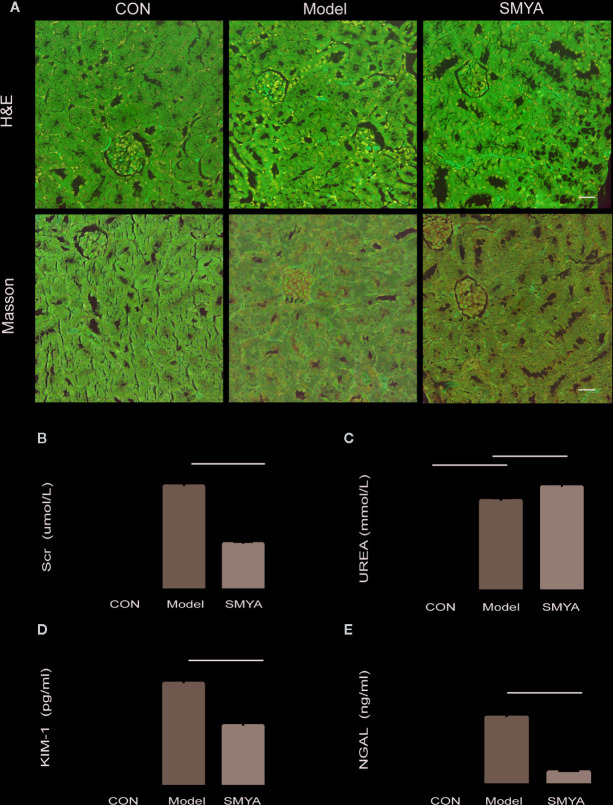
Results of histology and renal function. **(A, B)** HE staining and Masson staining, there was no significant change in the atherosclerosis model compared with the control in the HE staining. The atherosclerosis model and SMYA group had serious fibrosis and there was no significant difference between the model group and SMYA group in MASSON staining. **(B)** Serum Cr level, the serum Cr was significantly higher in the model group and decreased in the SMYA group. **(C)** Serum UREA level—there was a statistical significant difference among three groups, but the differences of mean values between any two groups had no clinical significance (<1.5mmol/L). **(D)** Urine KIM-1 level, SMYA decreased the urine KIM-1 significantly compared with the model. **(E)** Urine NGAL level, SMYA decreased the urine NGAL significantly compared with the model. ^∗^p < 0 05 and ^∗∗^p < 0 01.

HE staining and MASSON staining were conducted to observe histology changes. Results showed that there was no significant change in the atherosclerosis model compared with the control in the HE staining. In MASSON staining, results showed that fibrosis was serious in atherosclerosis model compared with that in the control, while there was on significant difference between model group and SMYA group (**Figure 1A**).

To examine if SMYA decoction could protect renal function, we detected the serum Cr, UREA and urinal KIM-1, and NGAL in mice of three groups. Results indicated that the serum Cr (**Figure 1B**) was significant higher in model group than that in blank group, and SMYA decoction could successfully decrease the serum Cr level to that of control. What is more, the results of urinal KIM-1 (**Figure 1D**) and NGAL (**Figure 1E**) showed the same trend as serum Cr. For serum UREA (**Figure 1C**), although statistical analysis told that there was statistical significant difference among the three groups, the differences of mean values between any two groups were smaller than 1.5mmol/L, which had no biological significance. Besides, histology study was also conducted. Both results of Masson and HE staining demonstrated that there was no significant difference among the three groups. All in all, the results above indicated that SMYA decoction have a good renal-protect function in atherosclerosis model.

### Renal Protection Function of SMYA Decoction May Not Be Related With Lowering Lipid Accumulation, Inflammation, And Oxidative Stress

**Figure 2:**
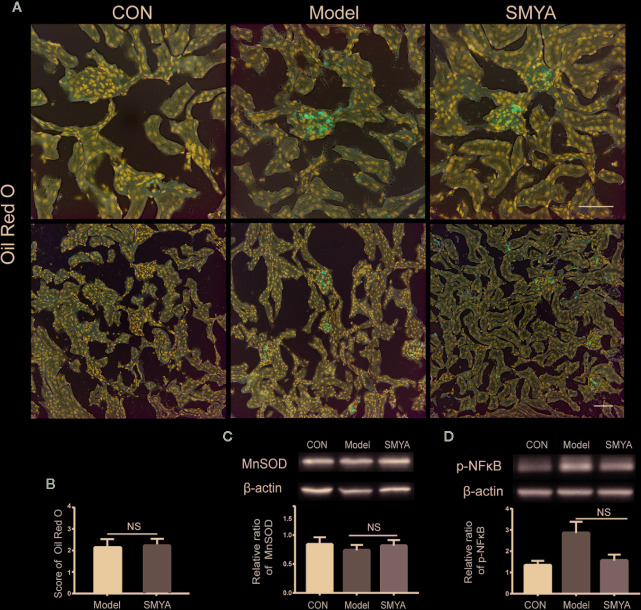
The lipid accumulation, p-NFκB, and SOD in kidney cortex tissue. **(A, B)** Lipid accumulation did not happen in the control group, while, in the model group and SMYA group, lipid accumulation was serious. There was no significant difference in lipid accumulation between model group and SMYA group. **(C)** SOD level in kidney cortex tissue. There was no significant difference among three groups. **(D)** p-NFκB level in kidney cortex tissue. There was no significant difference among three groups. Bars represent standard error of the mean (SEM). The representative blots were showed in this figure, and other original blots were submitted as **Supplementary Material**. NS means no significant difference vs. model.

We know that the lipids can accumulate in kidney tissue when hyperlipidaemia occurs. Lipid accumulation can cause renal damage, which is called lipid nephrotoxicity (Gai et al., 2019). In this study, we observed lipid accumulation in kidney tissue *via* oil red O staining. Results showed that the lipid mainly accumulated in the glomerulus. In the atherosclerosis model, lipid accumulation was serious, while no red lipid spot was observed in the control mice. The most important is that SMYA failed to alleviate the lipid accumulation (**Figures 2A**, **B**). It means that the renal protection function of SMYA decoction was not related with improving lipid accumulation. Except for lipid accumulation, p-NFκB and SOD in the kidney cortex tissue were also detected by WB. Results showed that there was no significant difference between SOD in atherosclerosis model and that in SMYA group (**Figure 2C**). As for p-NFκB, it demonstrated a downtrend in SMYA group, but it has no statistical significance compared with atherosclerosis model (**Figure 2D**).

### SMYA Decoction May Protect Renal Function Through Upregulating Autophagy-Mediated Degradation of Ubiquitinated Protein

**Figure 3:**
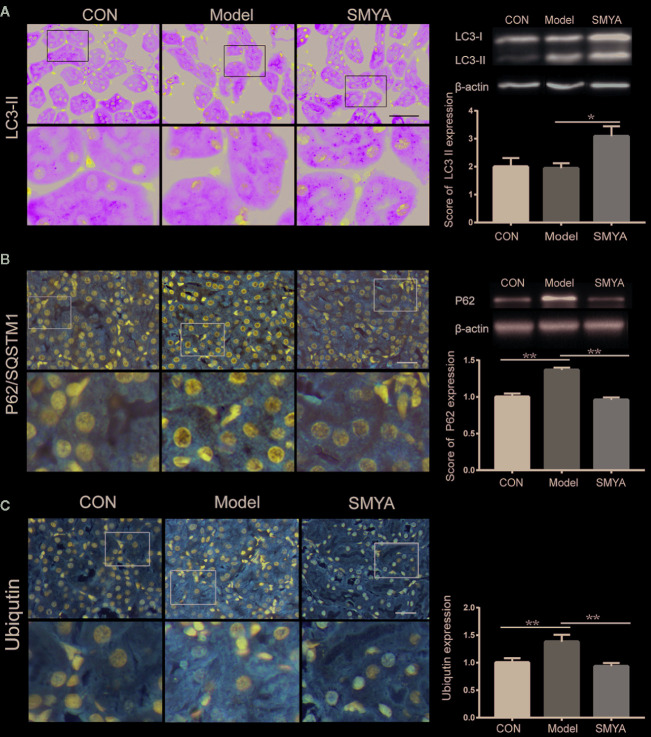
SMYA decoction upregulated autophagy-mediated degradation. **(A)** IF staining of LC3. More LC3 dots were observed in the SMYA group (n=7, 9, and 7 in the control group, model group, and SMYA group). **(B)** IHC staining of P62. More P62 was observed in the model group and declined in the SMYA group (n=6, 8, and 7 in control group, model group, and SMYA group). **(C)** IHC staining of UB (ubiquitinated protein). More UB was observed in model group and decreased in SMYA group (n=6, 9, and 8 in the control group, model group, and SMYA group). Bars represent standard error of the mean (SEM). The representative blots are shown in this figure, and other original blots have been submitted as **Supplementary Material**. ^∗^p < 0 05 and ^∗∗^p < 0 01.

In order to explore the mechanism of the renal-protect function of SMYA decoction, ubiquitinated protein was measured in the kidney tissue. Results demonstrated that more ubiquitinated protein accumulated in the kidney cells in model group compared the other two groups (**Figure 3C**). It meant that SMYA decoction improved the ability of degrading the ubiquitinated protein in kidney cells.

We know that autophagy is an important way to degrade the ubiquitinated protein (Kaniuk et al., 2007; Liu et al., 2016). Did the SMYA decoction promote the degradation of ubiquitinated protein *via* activating autophagy? To answer this question, the level of LC3 and P62 were measured with the method WB, IHC, and IF. The same as ubiquitinated protein, P62 increased in the model group and was decreased by SMYA decoction (**Figure 3B**). Results of LC3 illustrated that SMYA decoction could significant upregulate the express level of LC3 II (**Figure 3A**). These results indicated that SMYA decoction might upregulate the ubiquitinated protein degradation by activating the autophagy.

## Discussions

Because of the high-fat diet and sedentary lifestyle, more and more people get metabolic syndrome, which includes hyperlipidemia. We all know that hyperlipidemia can cause fatty liver and even liver cirrhosis. However, the harm of lipid nephrotoxicity also needs attention. Hyperlipidemia can lead to lipid abnormal accumulation in kidney tissue and cause injury on mesangial cells, podocytes, and proximal tubular cells (de Vries et al., 2014).

Even though the concept of lipid nephrotoxicity was proposed 47 years ago (Moorhead et al., 1982), the mechanism of lipid nephrotoxicity is still not clear. Some studies indicated that lipid-induced renal damage was related to inflammatory pathways and over production of ROS (Gyebi et al., 2012; Kennedy et al., 2013; Gao et al., 2014). Hyperlipidemia contributes to systemic oxidative stress. Moreover, TNF (tumor necrosis factor) and IL1β can increase ROS level in the kidney (Ruan et al., 2009). The increased ROS generation can cause oxidative damage to lipid and lipid peroxidation was the first step of oxidized LDL, which can accumulate in the kidney and lead cytotoxic effects (Ruan et al., 1999). Besides, Oxidative stress can activate the NFκB pathway, which has a close relationship with inflammation and the progression of CKD (Janabi et al., 2000; Guijarro and Egido, 2001). What is worse, no drug that has been clearly proved to be effective in preventing lipid nephrotoxicity. Even though statins can significantly reduce the blood lipid, there is no proof to prove of their extra efficacy in protecting the renal function (Baigent et al., 2011).

Fortunately, we have alternative therapy to treat lipid nephrotoxicity. Traditional Chinese medicine (TCM) is widely used in clinic. SMYA decoction is a famous ancient prescription, and it is still widely used by TCM doctors in treating many diseases, including preventing lipid nephrotoxicity. In this study, we observed that SMYA decoction can effectively reduce the Scr, urinal KIM-1, and NGAL in the atherosclerosis model, which indicates that SMYA has the function of protecting renal function. We know that inflammatory pathways and over production of ROS play an important role in the lipid nephrotoxicity. Does SMYA protect kidney function through regulating inflammation or ROS? The results in this study indicate that SMYA cannot regulate p-NFκB and ROS. It even cannot alleviate the lipid accumulation. Results above indicate that SMYA acts independently on regulating lipid metabolism, inflammation, and oxidative stress.

A study has proven, however, that mitochondrial damage has happened in the high fat diet-induced insulin resistance mice (Yuzefovych et al., 2013). Mitochondrial damage happening means that excessive damaged protein accumulates, which will be ubiquitinated for further degradation. If the production of ubiquitinated protein exceeds its degradation, protein homeostasis and cell cycle will be disrupted (Hammerling and Gustafsson, 2014; Liu et al., 2016). We can therefore hypothesize that ubiquitinated protein maybe accumulated in hyperlipidemia subjects, which cause renal injury. In this study, results of UB detection showed that the level of UB increased obviously in the atherosclerosis model mice and SMYA could effectively decrease the UB. But how SMYA degraded the accumulated UB? As we know, autophagy is an important ubiquitinated protein degradation mechanism (Liu et al., 2016; Forrester et al., 2018). P62 plays an important role in delivering the ubiquitinated protein to autophagosome and then degraded by the lysosome (Liu et al., 2016). LC3 is the most widely monitored autophagy-related protein, which is an important protein in the formation of autophagosome and cargo selection (Galluzzi and Green, 2019). In this study, we found that SMYA could reduce the level of P62 and increase the level of LC3. These results indicate that SMYA may improve ubiquitinated protein degradation *via* activating the autophagy flux and its renal-protect function should be related to regulation of the autophagy-mediated ubiquitinated protein degradation.

In this study, we found that SMYA decoction, a famous ancient decoction of TCM, could protect renal function in a atherosclerosis model and might be related to the autophagy-mediated degradation of ubiquitinated protein. The result of this study is helpful for the treatment of kidney disease caused by lipotoxicity. In order to make the mechanism clearer, better hyperlipidemia models and vitro experiments are need in the future research.

## Data Availability Statement

All datasets generated for this study are included in the article/**Supplementary Material**.

## Ethics Statement

The animal study was reviewed and approved by Animal Ethics Committee of Beijing University of Chinese Medicine (NO.BUCM-4-2015071701-3001).

## Author Contributions

Z-BZ, KS, and W-JH acquired the data. HL, HY, Y-QB, K-TG, R-BY, W-JLo, and C-HX contribute their efforts in animal keeping and sacrifice. BN and W-JLi designed the study.

## Conflict of Interest

The authors declare that the research was conducted in the absence of any commercial or financial relationships that could be construed as a potential conflict of interest.
