# Supplementary material for: Si-Miao-Yong-An (SMYA) Decoction May Protect the Renal Function Through Regulating the Autophagy-Mediated Degradation of Ubiquitinated Protein in an Atherosclerosis Model
Source: Front Pharmacol. 2020 Jul 2;11:837. doi: 10.3389/fphar.2020.00837 (PMC7343850; doi:10.3389/fphar.2020.00837)
Supplement: Supplementary file 1 [file DataSheet_1.pdf]

	CON	Model	SMYA
GLU（mmol/L）	7.0889± 0.37602	7.5782±0.4194	4.1478±0.49328**
TC（mmol/L）	2.8922±0.294	28.6918±0.68431	45.6100±4.06087**
TG（mmol/L）	1.5900±0.2184	1.4327±0.14023	1.6489±0.20013
HDL（mmol/L）	1.6000±0.17125	1.0900±0.0627	0.4533±0.049527**
LDL（mmol/L）	0.4433±0.06491	7.2327±0.46574	15.6444±0.22509**
ALT（mmol/L）	44.8778±5.26286	38.4636±7.7269	68.1889±24.835
AST（mmol/L）	80.2444±10.85328	87.4818±14.0521	81.6333±10.56724

** Compared with Model group, P＜0.01.
