# Supplementary figures and images for: Si-Miao-Yong-An (SMYA) Decoction May Protect the Renal Function Through Regulating the Autophagy-Mediated Degradation of Ubiquitinated Protein in an Atherosclerosis Model
Source: Front Pharmacol. 2020 Jul 2;11:837. doi: 10.3389/fphar.2020.00837 (PMC7343850; doi:10.3389/fphar.2020.00837)

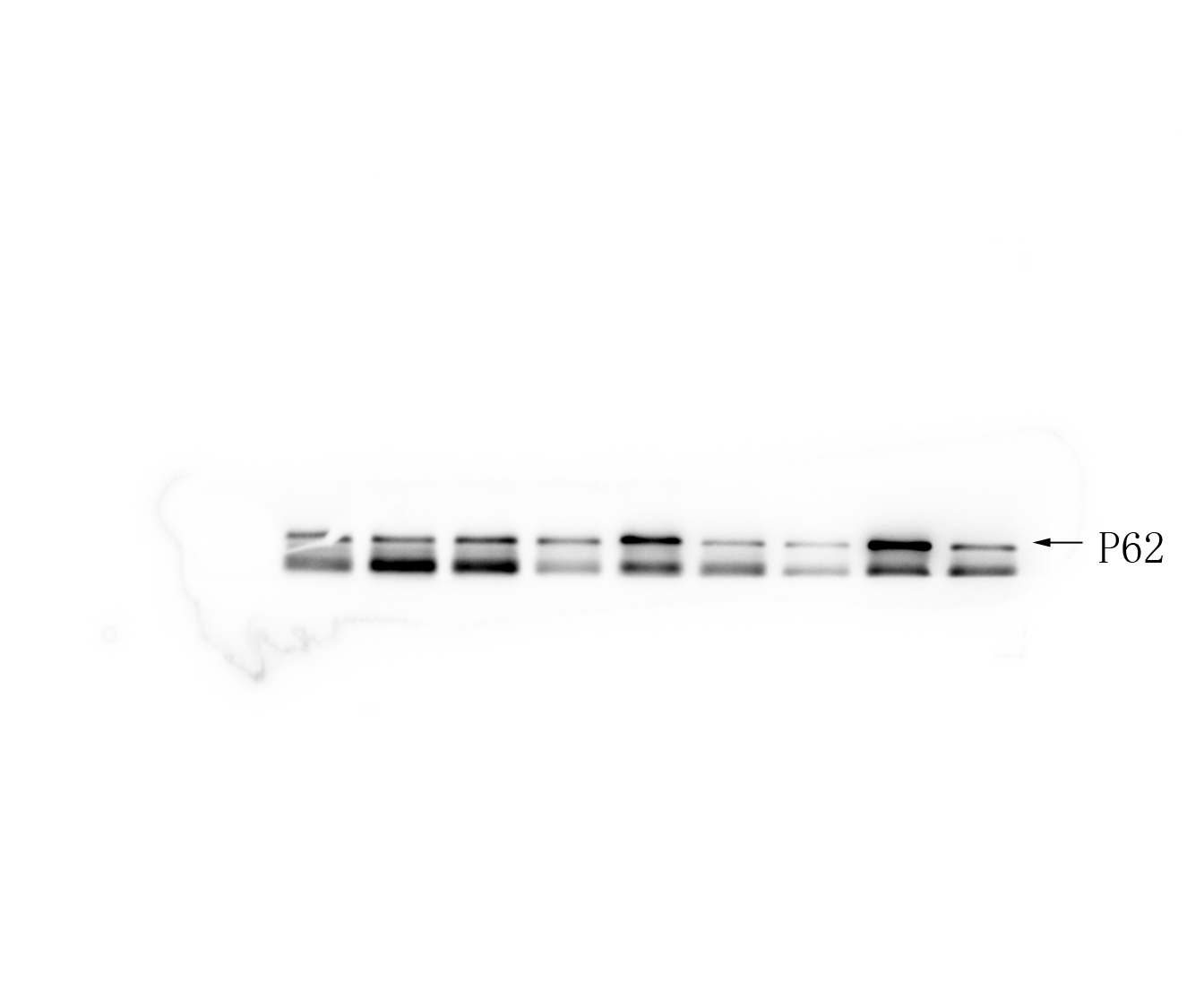

Supplement: Supplementary file 2 [file Image_1.tif]

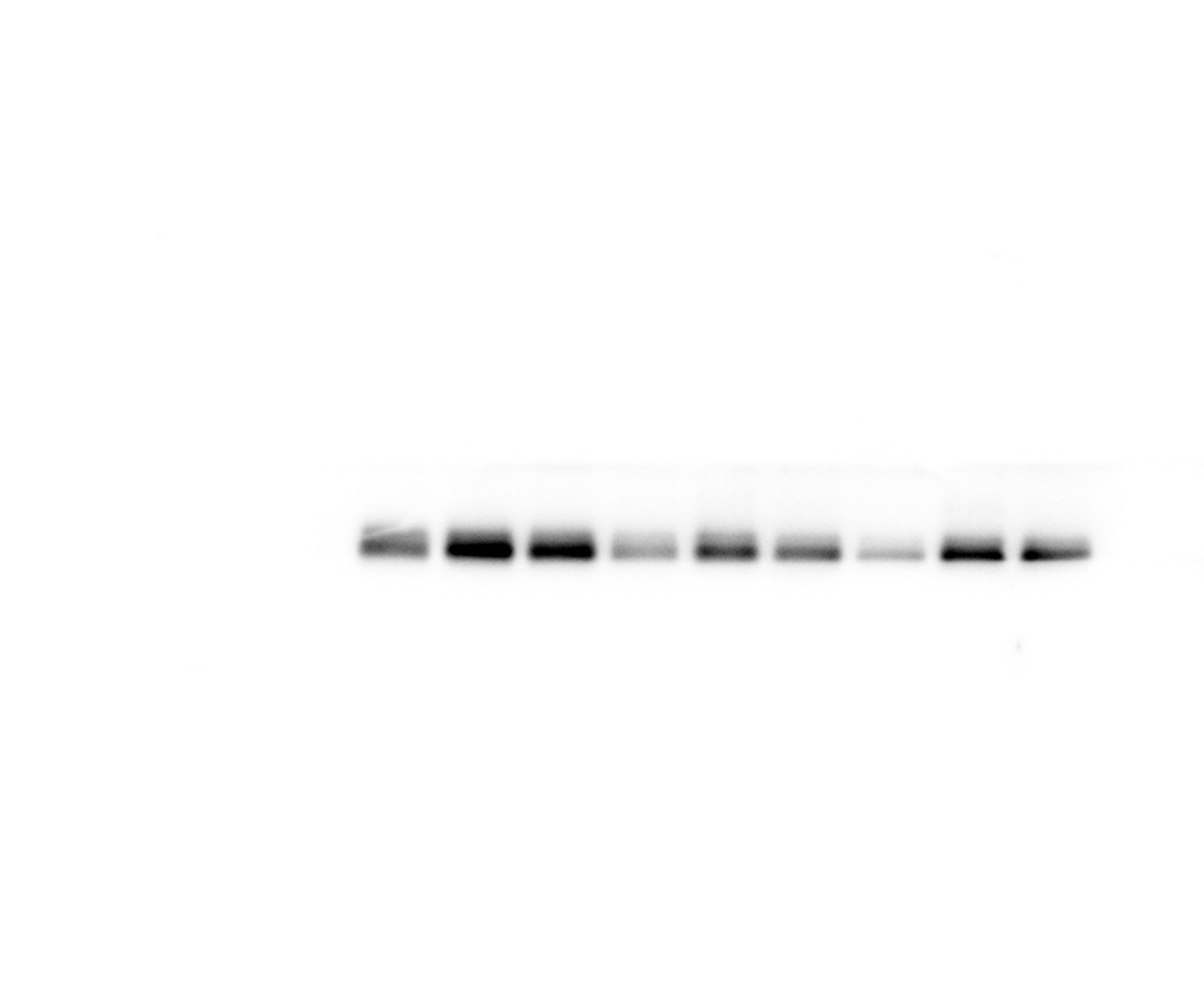

Supplement: Supplementary file 3 [file Image_2.tif]

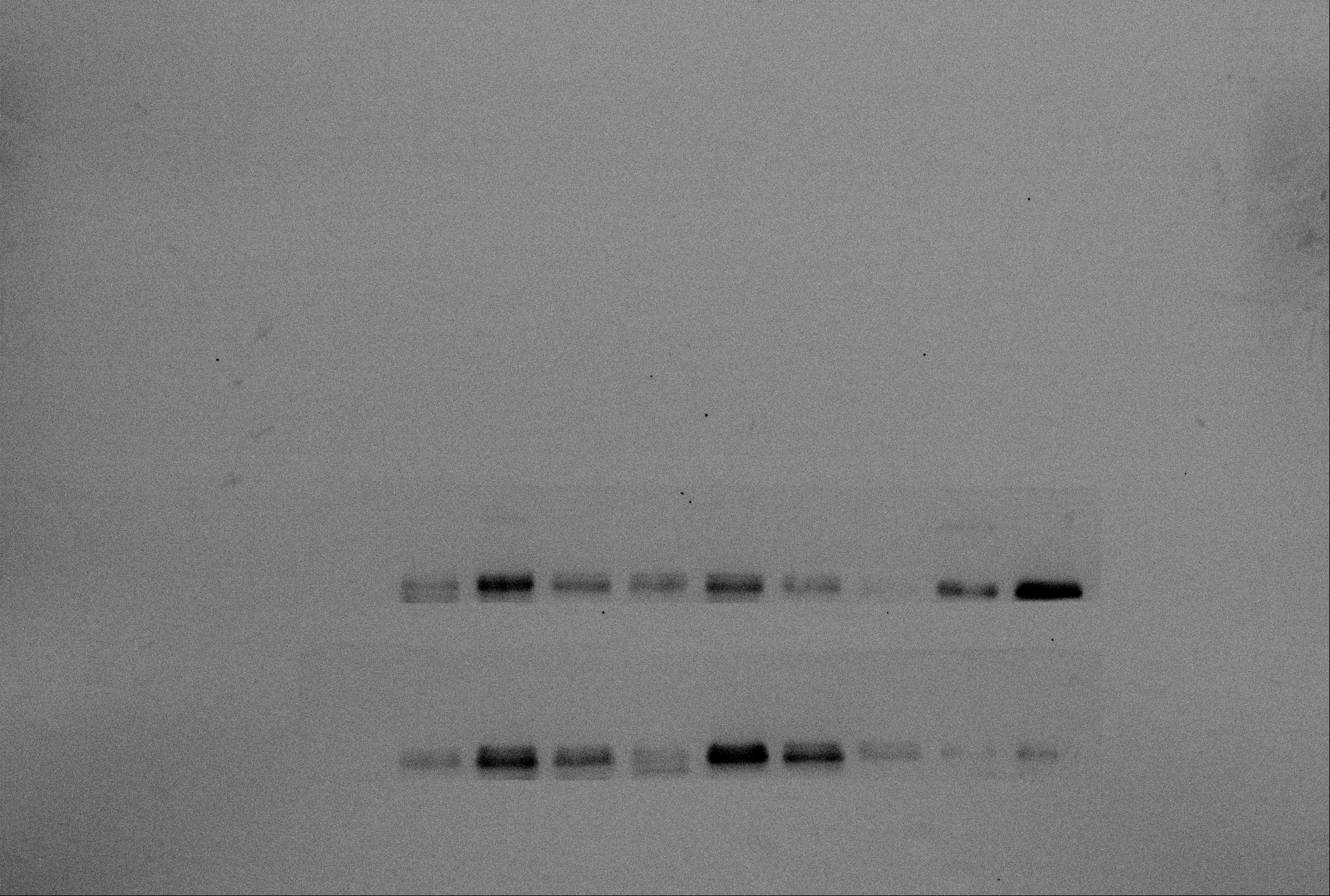

Supplement: Supplementary file 4 [file Image_3.tif]

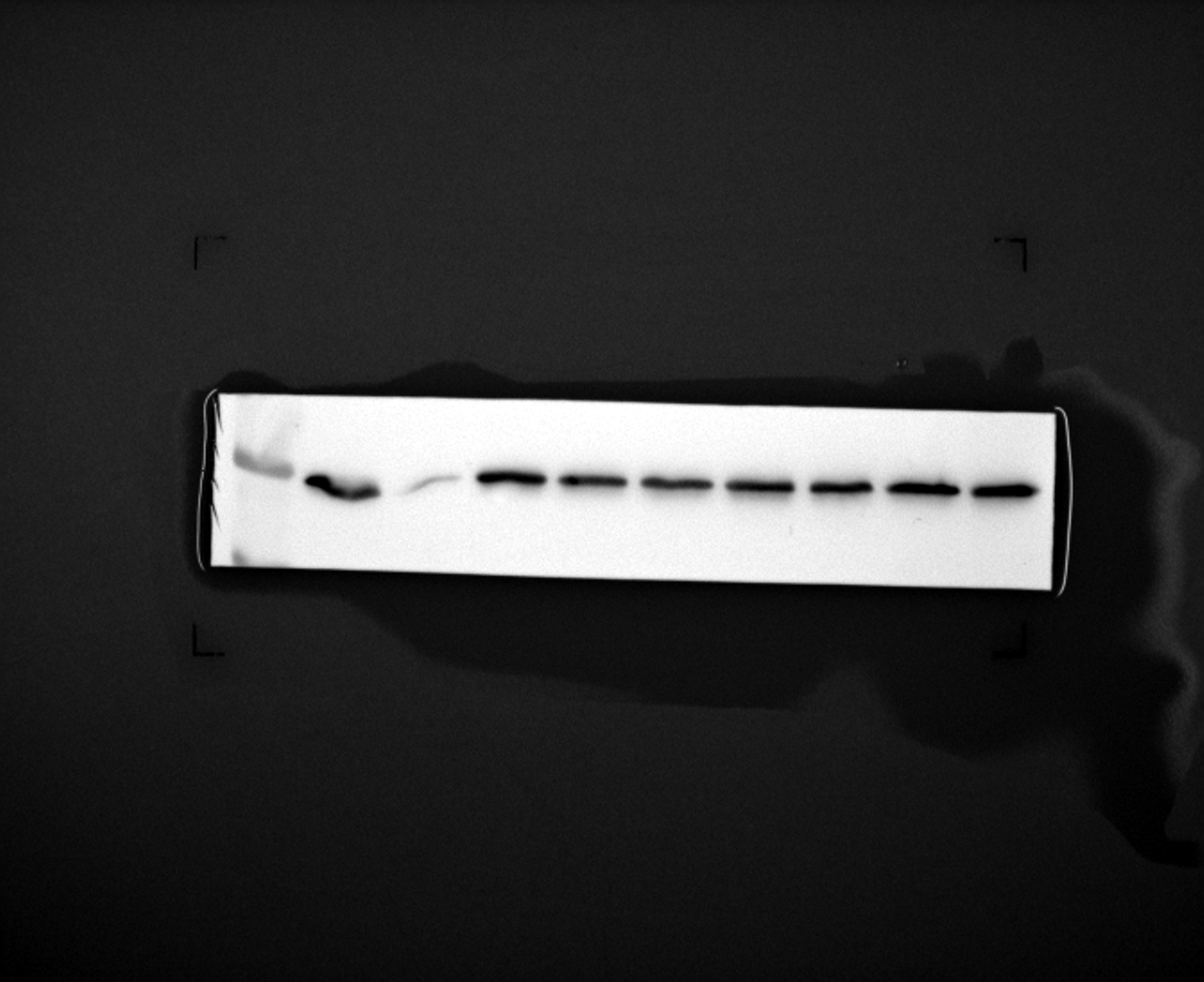

Supplement: Supplementary file 5 [file Image_4.tif]

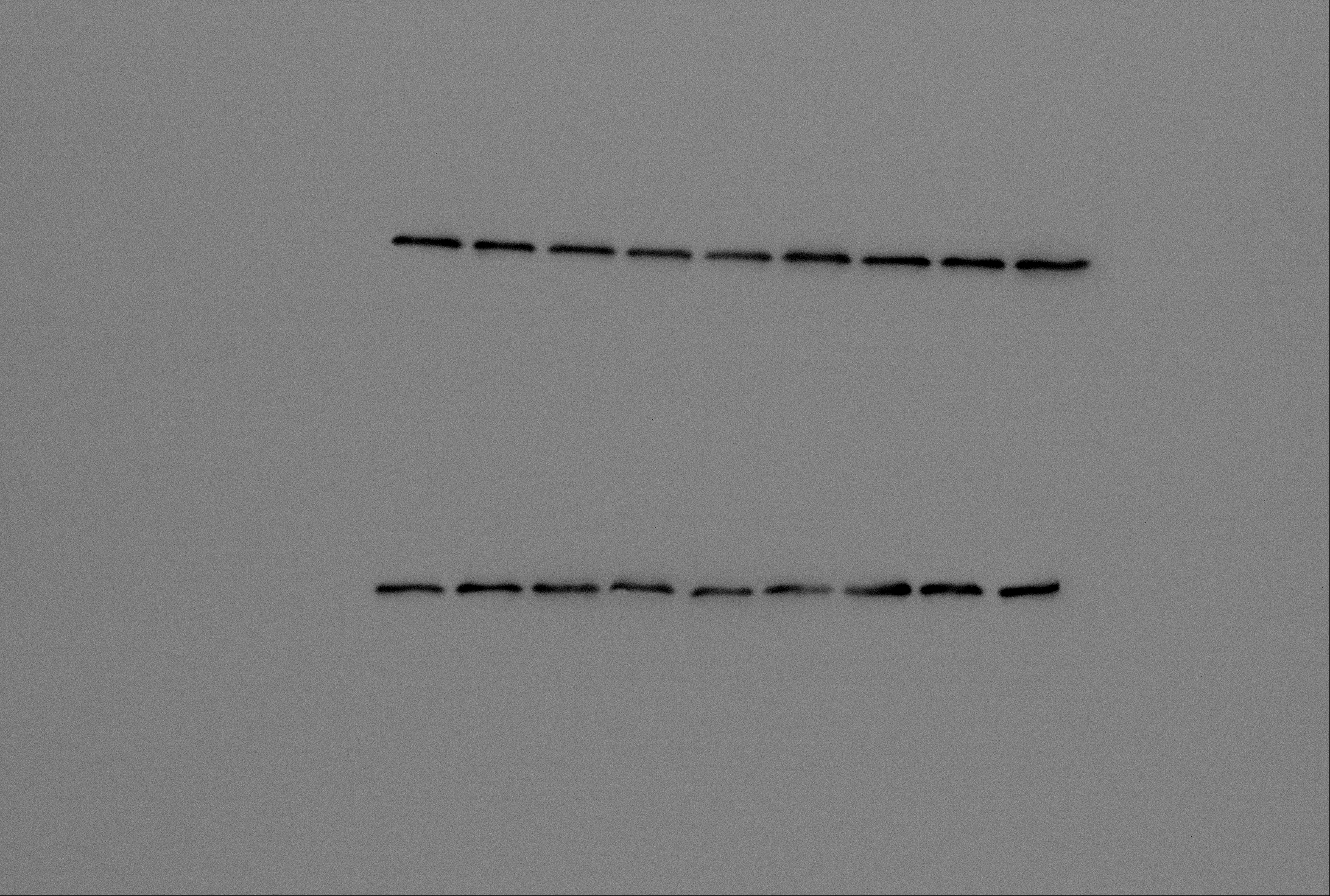

Supplement: Supplementary file 6 [file Image_5.tif]
